# Multiplex gene editing drives revolution in crop breeding: overlaid editing of multiple genes and customization of complex traits

**DOI:** 10.1007/s44307-026-00099-7

**Published:** 2026-02-19

**Authors:** Jieni Lin, Hanipa Hazaisi, Yuefeng Guan, Mengyan Bai

**Affiliations:** 1 Guangdong Provincial Key Laboratory of Plant Adaptation and Molecular Design, College of Life Sciences, Guangzhou University, Guangzhou, 510006 China; 2 Ili Agricultural Science Institute, Yining, 835100 China

**Keywords:** CRISPR/Cas9, Multiplex genome editing, Crop breeding, Gene editing strategies

## Abstract

Modern agriculture currently demands higher standards for the simultaneous improvement of crop yield, quality and stress resistance. However, traditional crop breeding methods can no longer meet the needs of modern agricultural development. Improving a single trait is no longer sufficient to meet the multifaceted demands of modern agricultural production and consumer expectations. Multiple traits breeding has increasingly become a key objective in current crop breeding. Over the past decade, CRISPR/Cas9-based multiplex genome editing (MGE) has enabled efficient pyramiding and precise regulation of multiple traits via targeted editing of multiple gene loci, revolutionizing crop breeding. In this review, we briefly describe the core CRISPR/Cas-based MGE strategies and technical workflows, and thoroughly discuss the practical outcomes of MGE applications in various fields, such as enhancing crop stress resistance, increasing yield and improving quality. This review aims to provide a summary and theoretical reference for crop breeding, as well as open up new ideas for achieving different breeding goals.

## Introduction

Driven by the continuous growth of the global population and the upgrading of living standards, modern agriculture imposes higher requirements on crop breeding, including multi-dimensional trait improvement such as yield enhancement, flavor quality optimization and nutritional component enhancement. Consequently, multiple traits pyramiding has become a key direction in crop genetic improvement. Traditional hybrid breeding has long been the primary method for multi-gene pyramiding, which relies on genetic recombination to stack multiple traits (Collard and Mackill 2008). However, traditional hybrid breeding has critical limitations for multi-gene pyramiding. It requires 10–15 years per cycle, with low screening efficiency that hinders rapid development of new varieties for agricultural production. Moreover, constrained by genetic linkage effects, genes controlling different favorable traits are often located on the same chromosome or in the same functional module, which significantly increases the difficulty of precise pyramiding and easily leads to genetic drag. Meanwhile, the randomness of genetic recombination during hybridization prevents the targeted regulation of multi-gene networks, making it difficult to create rare or novel combinations of favorable traits that do not exist in nature. From the perspective of genetic mechanisms, crop yield is not only directly regulated by genes related to growth and development, but also exhibits complex synergistic interactions with gene networks of multiple pathways such as disease resistance, pest resistance and stress tolerance (Mickelbart et al. 2015). In contrast, the genetic basis of crop quality traits is more complex, involving multiple aspects such as nutritional components, taste and appearance. Traditional breeding techniques are mostly limited to single-trait improvement, making it difficult to achieve the coordinated enhancement of complex traits (e.g., yield, quality and stress resistance), which results in an inherent gap with the demand for optimizing crop comprehensive traits in modern agriculture.

Against this background, the CRISPR/Cas system, as the third-generation gene editing technology, has become a core tool in the field of crop genetic improvement, owing to its characteristic of single guide RNA (sgRNA)-guided, nuclease-mediated specific recognition and cleavage of double-stranded DNA (dsDNA) (Jinek et al. 2012; Hsu et al. 2013; Nishimasu et al. 2014; Gao 2021; Wang and Doudna 2023). The CRISPR/Cas system is a programmable RNA-guided nuclease platform: sgRNA forms a ribonucleoprotein complex with Cas endonuclease, which recognizes target dsDNA via sgRNA-target base pairing and recognition of the protospacer adjacent motif (PAM, typically NGG), and then mediates site-specific double-strand breaks (DSBs) (Jinek et al. 2012; Doudna and Charpentier 2014). These DSBs trigger cellular DNA repair mechanisms: predominantly non-homologous end joining (NHEJ), an error-prone pathway, and homology-directed repair (HDR), a template-dependent precise repair pathway. For gene knockout, NHEJ-induced small insertions or deletions (indels) often disrupt the target gene’s open reading frame via frameshift mutations or premature termination codons, abrogating functional protein expression (Mali et al. 2013; Q. Zhang et al., 2018). With the continuous iteration of CRISPR technology, novel multiplex genome editing tools have been continuously developed (Jinek et al. 2012; Cong et al. 2013; Feng et al. 2013; Ma et al. 2015; Char et al. 2017), significantly improving editing efficiency and targeting scope. As an important extension of the CRISPR/Cas system, multiplex genome editing technology has a core advantage: it enables efficient editing of multiple gene loci via a single vector (single transformation) (Abdelrahman et al. 2021). Multiplex genome editing can coordinate the expression levels of key genes across pathways, enabling stacked editing and precise customization of different traits. It promotes the transformation of plant breeding from experience-dependent to design-driven, provides a revolutionary technology for breaking through the bottlenecks of traditional breeding, and highlights its unique advantage in crop breeding: the ability to edit multiple target genes without altering unrelated genes and to precisely regulate multiple target traits.

The emergence of multiplex genome editing technology has overcome the limitations of single-target manipulation in early gene editing technologies, advancing crop genetic improvement from single-gene modification to synergistic regulation of multi-gene networks (Devi et al. 2022). Unlike the passive mode of traditional breeding that relies on random recombination, multiplex genome editing can simultaneously edit multiple genes distributed across different chromosomes or functional modules through the precise targeting capability of the CRISPR/Cas system. The breakthroughs of multiplex genome editing are reflected in three aspects. First, innovation in editing efficiency shortens the multi-trait pyramiding cycle to 2–3 years. For instance, by optimizing a small-scale mixed-pool technical pathway, plasmids containing 3–5 target function-related genes were mixed and stably transformed, resulting in the development of an efficient technical route for creating multi-gene mutation populations (Bai et al. 2020). Second, enhanced targeting precision, which leverages the targeting specificity of CRISPR/Cas system, avoids genetic linkage interference. For example, simultaneous editing of 8 gliadin genes (including *Gli-γ* and *Gli-ω*) in wheat enables targeted reduction of allergen content without affecting other agronomic traits (Sánchez-León et al. 2024). Third, multiplex gene editing advances from single pathway regulation to synergistic optimization of multiple metabolic networks. For example, simultaneous editing of the carotenoid synthesis gene *PSY1*, flavonoid synthesis gene *MYB12*, and chlorophyll degradation gene *SGR1* in tomatoes achieves systematic improvement of fruit color and nutritional components (Yang et al. 2023).

## Overview of research on multiplex genome editing technology

### Development context of multiplex genome editing technology

The expansion of genome editing scope is associated with the demand for crop polygenic trait improvement. The initial stage of multiplex genome editing originated from the maturation of single-gene editing tools, gradually achieving a breakthrough from single-target intervention to multi-target coordinated regulation. Genetic redundancy is an obstacle to trait improvement in crops. Single-gene mutations often lead to inconspicuous phenotypic changes due to functional redundancy. Specifically, the functional compensation effects of homologous family genes usually make the performance of target traits not significant after single-gene mutation. In contrast, multiplex genome editing technology can effectively reveal the target phenotype through synchronously editing multiple members of a gene family. For example, in wheat, only the simultaneous mutation of three *TaMLO* homoeoalleles (A, B and D genome copies) mediated by TALEN could endow plants with broad-spectrum resistance to powdery mildew (Wang et al. 2014). Plants with only a single copy knocked out failed to exhibit stable resistance due to functional compensation from other homoeologous genes. In dicotyledonous plants, durable disease resistance similarly relies on multi-gene mutations. For instance, the *AtMLO2/6/12* triple mutant of *Arabidopsis thaliana* can significantly enhance pathogen resistance (Consonni et al. 2006). It should be noted that TALEN technology is not suitable for multiplex gene editing due to its complex vector construction and low targeting efficiency in crops.

The development of the CRISPR/Cas system has enriched the toolkit of multiplex gene editing technology. Simultaneous editing of multiple target loci with two sgRNAs via a single vector was first achieved in *Arabidopsis thaliana* and rice (Feng et al. 2013; Lu et al. 2017). Among the components, *Cas9* was expressed under the control of the *35S* promoter to ensure stable synthesis in plant cells, while sgRNAs were driven by plant-specific *U6*/*U3* promoters. This design was adapted to the plant transcriptional mechanism and improved targeting efficiency, successfully verifying the feasibility of CRISPR/Cas9-mediated multiplex gene editing in these two model plants.

A key milestone in the development of multiplex gene editing technology lies in the innovative optimization of vector design systems. Researchers have developed a streamlined genome editing tool by linking tRNA and sgRNA to form compact tandem repeats (Xie et al. 2015). The tandem arrangement of tRNA-sgRNA structures enables the simultaneous production of multiple sgRNAs, which then guide the Cas9 protein to precisely cleave several targeted DNA sequences. When tested in rice across four gene loci (with 2, 4, and 8 sgRNAs), the construct containing two sgRNAs showed only slightly higher efficiency compared to polycistronic structures with four and eight sgRNAs. To date, the highest reported record in crop breeding is the simultaneous editing of 13 target sites in rice (Hao et al. 2025). Overall, the efficiency of gene editing and the number of editable sites in monocotyledonous plants are superior to those in dicotyledonous plants. In summary, multiplex gene editing technology has enhanced the phenotypic performance of target traits (Z. Li et al., 2021), broken the negative correlation among different crop agronomic traits (Zhong et al. 2024), and enabled the directional customization of complex phenotypes in crops (T. Li et al. 2018a).

### Types of multiple sgRNA co-expression systems

**Fig. 1:**
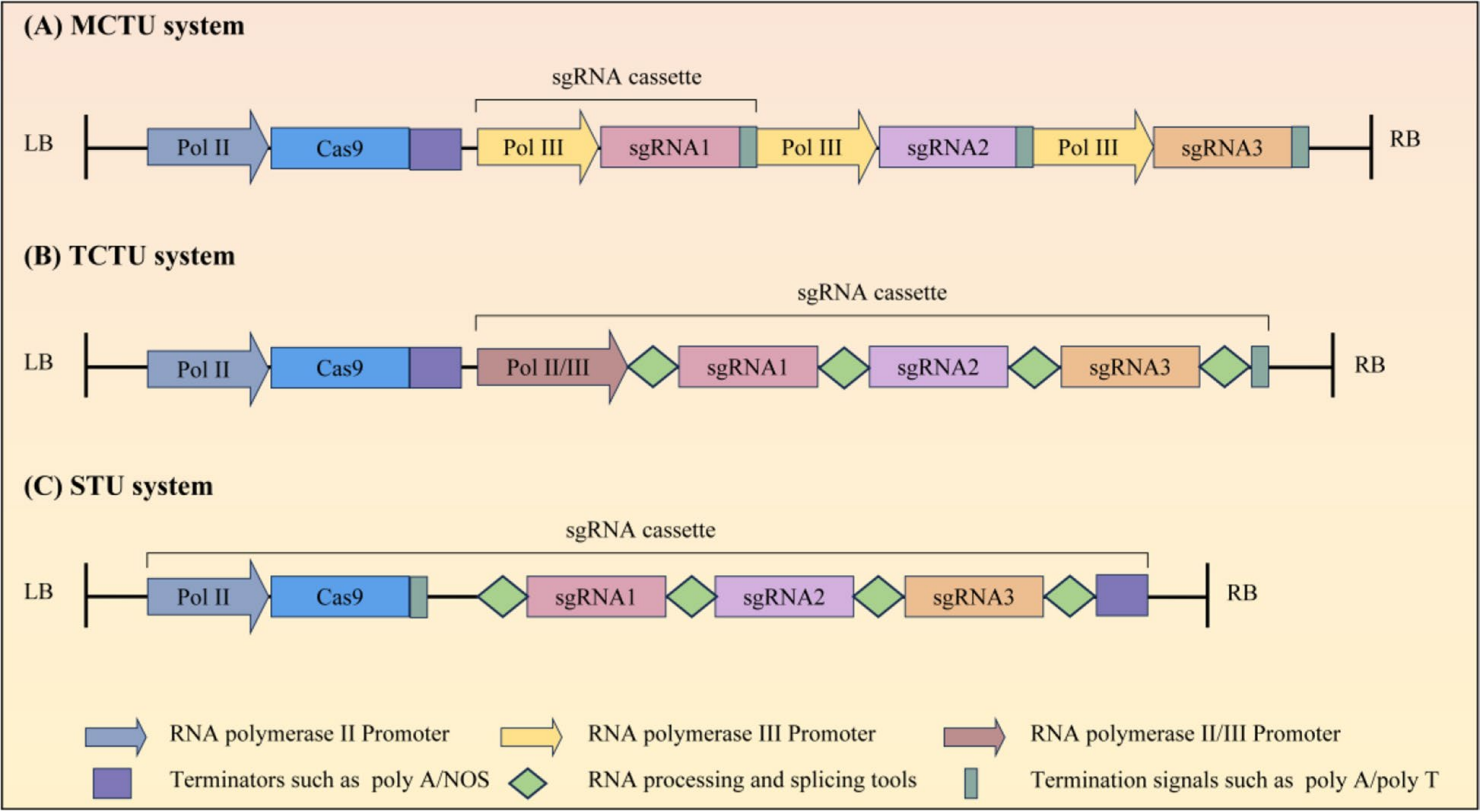
Schematic of three multiple sgRNA co-expression systems for CRISPR/Cas9-mediated multiplex genome editing in plants. **A** MCTU system: Cas9 is expressed by a Pol II promoter, with each sgRNA driven by an independent Pol III promoter. **B** TCTU system: Cas9 is expressed by a Pol II promoter, and all sgRNAs are transcribed as a single polycistronic precursor. RNA processing elements cleave the precursor into mature sgRNAs. **C** STU system: Cas9 and sgRNAs are fused into one Pol II-driven transcript, processed post-transcriptionally

In CRISPR/Cas-mediated multi-gene editing, multiple sgRNAs co-expression systems are the core to achieving efficient simultaneous editing of multiple target loci and feasible vector delivery. Three main types have been developed, each with distinct structural designs and application characteristics (Fig. 1) (Zhang et al. 2019).

The Multi-Transcriptional Unit (MCTU) system employs an independent RNA polymerase III (Pol III) promoter (e.g., *U6*, *U3*) to drive the expression of each sgRNA, with the *Cas9* expression cassette physically separated from the sgRNA array (Fig. 1A) (Xing et al. 2014; Zhang et al. 2016). This modular design enables precise control of the editing efficiency for individual targets by selecting promoters of varying strengths, adapting to loci with different genomic accessibility. However, tandem repetition of homologous Pol III promoters increases the risk of plasmid recombination during replication, and the cumulative length of multiple promoters and terminators results in larger vector size, limiting its use in size-constrained delivery systems (Ding et al. 2020). It remains preferred for editing 2–4 loci in monocots (e.g., rice, maize) due to its simple design and high compatibility (Xing et al. 2014; Ma et al. 2015). In contrast, the Twin-Transcriptional Unit (TCTU) system decouples *Cas9* and sgRNA expression into two independent transcriptional units: a single Pol II promoter (e.g., *Ubiquitin*, *ZmUBQ*) drives the expression of *Cas9* fused with a nuclear localization signal (NLS) for nuclear targeting, while multiple sgRNAs are transcribed as a single precursor transcript under the control of a single Pol II or Pol III promoter. Interspersed cleavage elements (e.g., tRNA, Csy4 ribozyme, Hammerhead ribozyme) mediate the post-transcriptional processing of the precursor transcript to release mature sgRNAs (Fig. 1B) (Zhang et al. 2019; Carrijo et al. 2021). Among these, the tRNA-sgRNA strategy is the most widely used in plants: it leverages the endogenous tRNA maturation machinery (RNase P and RNase Z) to recognize conserved tRNA structural motifs, precisely cleaving the tRNA-sgRNA junctions to release functional, mature sgRNAs without relying on exogenous factors (Canino et al. 2009). For example, in rice, a tRNA-sgRNA array targeting three independent genes achieved a > 50% simultaneous mutation efficiency, and the absence of promoter repetition reduced the risk of vector recombination (Xie et al. 2015). Its broad applicability stems from its structural simplicity and compatibility with most plant species. In contrast, the Csy4 ribozyme variant relies on co-expressed Csy4 endonuclease to cleave specific recognition sequences between sgRNAs, offering high cleavage efficiency but increasing vector complexity due to the need for exogenous nuclease expression (Haurwitz et al. 2010; Liu et al. 2019). By comparison, Hammerhead ribozymes exhibit the lowest self-cleavage efficiency. Based on these components, researchers have developed a modular, broadly applicable plant multiplex genome editing toolkit based on the TCTU system, which is capable of supporting simultaneous editing at up to eight target sites (Čermák et al. 2017). The TCTU system balances editing efficiency and vector compactness, making it versatile for 4–8 loci editing in plants, and is the most commonly used platform for medium-scale multi-gene editing. The Single Transcriptional Unit (STU) system fuses Cas9 and sgRNA into one transcriptional unit, where sgRNAs are linked to Cas9 via flexible RNA linkers (e.g., introns) for independent processing (Fig. 1C) (Tang et al. 2016). It has the most compact vector structure, suitable for protoplast transient transformation and gene stacking (Bai et al. 2022).

### Optimization strategies of multiplex gene editing efficiency

The achievement of multi-gene editing relies on efficient multi-sgRNA expression systems, and its technical development has undergone a critical transition from multi-vector mixed delivery to single-vector integration. Early research on multi-gene editing focused on the pooled CRISPR method (Berman et al. 2025; Jacobs et al. 2017; Lu et al. 2017; Meng et al. 2017). For example, when constructing nodulation and nitrogen fixation-related soybean mutagenesis populations, researchers mixed single-sgRNA vectors targeting nodulation and nitrogen fixation-related genes, successfully obtaining a series of new germplasms such as the *ric1a/2a* double mutant with increased nodule numbers (Bai et al. 2020). However, this early mixed vector strategy had key limitations. First, the number of editable loci was limited to 2–4, as the co-transformation efficiency in crop cells dropped sharply when the number of vectors exceeded 4, which failed to meet the needs of pathway-level multi-gene editing (Bai et al. 2020; Berman et al. 2025). Second, gene redundancy in polyploid crops further reduced the effective editing rate of the pooled CRISPR method (Cheng et al. 2025). These shortcomings drove the shift to single-vector multi-sgRNA expression systems, which integrate all sgRNA units into a single vector. By ensuring the co-delivery of all targets through unified promoter regulation or element tandem arrangement, these systems avoid the issues of multi-vector competition and random integration. Currently, the MCTU system is the most widely used and is primarily applied in monocot plants, where multiple sgRNAs are mostly driven by Pol III promoters. These promoters exhibit the following characteristics: (1) specifically initiating the transcription of short non-coding RNAs; (2) no requirement for poly(A) tail modification of transcription products; (3) strict base preference for transcription initiation (A for U3, G for U6) (Zhou et al. 2023). An MCTU-based CRISPR/Cas9 vector containing six sgRNAs was constructed by utilizing the characteristics of this system, targeting six *PYL* genes among the 14 members of the abscisic acid (ABA) receptor gene family in *Arabidopsis thaliana*. Six-gene mutants were obtained from 15 T_1_ generation plants, verifying the practicality of this system (Zhang et al. 2016).

Most multiplex genome editing studies in monocots adopt the MCTU and TCTU systems (Table 1), with applicability and optimization strategies varying by species: as a model monocot plant with a well-established endogenous processing system, rice uses the MCTU system as the first choice for large-scale trait pyramiding editing, combined with Pol III promoters to support the tandem arrangement of up to 16 sgRNAs (Wei et al. 2024), and Cas9 and sgRNAs can also be expressed as two separate units, with mature sgRNAs stably released via the rice endogenous tRNA processing machinery (Huang et al. 2024). Wheat, a polyploid monocot with a repeat-rich genome and a high risk of transgene silencing, is also most suitable for the MCTU system (Sánchez-León et al. 2024). Notably, as Pol III promoters are poorly characterized in many plant species and only suitable for short transcripts, using Pol II promoters to drive multiple sgRNAs is a preferable strategy for multiplex editing in crops with uncharacterized Pol III promoters, and certain Pol II promoters exhibit higher editing efficiency than Pol III promoters in plants (Tang et al. 2016; Čermák et al. 2017). For the tRNA-sgRNA tandem system, the main challenge is the small number of sgRNAs that can be tandemly arranged in a single vector. To address this issue, a three-element combinatorial expression system (tRNA-sgRNA-Ribozyme) was innovatively developed, achieving efficient tandem arrangement of 12 sgRNAs in rice for the first time, with an editing efficiency of 30% (Wei et al. 2024).

**Table 1:** Lists of multiplex genome editing strategies in crops

Editing strategy	Crops	Promoter of sgRNAs	Numbers of sgRNAs	Reference
MCTU	Rice	*OsU6a*	6	(Zeng et al. 2020)
	Rice	*OsU6a/OsU6b/OsU6c/OsU3m*	8	(Usman et al. 2020)
	Rice	*OsU3/OsU6a/OsU6b*	3	(Zhou et al. 2022)
	Rice	*OsU3*	3	(Zhang et al. 2024)
	Rice	*OsU6a/OsU6b/OsU6c/OsU3*	13	(Hao et al. 2025)
	Wheat	*TaU6*	2	(Sánchez-León et al. 2024)
	Soybean	*GmU3/GmU6*	4	(Zhang et al. 2020)
	Soybean	*AtU3b/AtU6-1/AtU3d/AtU6-29*	15	(Cheng et al. 2023)
	Rice	*aU3/aU6a/aU6b*	16	(Wei et al. 2024)
	Rice	*OsU3*	5	(He et al. 2024)
TCTU	Rice	*OsU3*	4	(Huang et al. 2024)
	Soybean	*GmU6*	4	(Cao et al. 2022)
	Tomato	*SIU6*	6	(Yang et al. 2023)
	Tomato	*CmYLCV*	6	(T. Li et al. 2018a)
STU	Rice	*pro Pol II*	2	(Tang et al. 2016)
	Soybean	*GmUBQ*	4	(Cao et al. 2022)
	Soybean	*GmUbi3*	3	(Carrijo et al. 2021)
	Soybean	*pM4*	5	(Lin et al. 2024)

For dicot plants, the STU system is more applicable due to the higher risks of transgene silencing and vector recombination in this group, with soybean as a typical example: as a dicot legume with low tolerance to vector redundancy and promoter repetition, soybean is highly suitable for genome editing via the STU system (Carrijo et al. 2021; Cao et al. 2022; Lin et al. 2024), in which Cas9 and sgRNAs are linked by poly(A) sequences, and the editing mode of sgRNA processing via endogenous tRNAs is the most common; driven by soybean endogenous Pol II promoters, this system can support the editing of up to 8 sgRNAs (Bai et al. 2024). In general, dicots necessitate the priority selection of species-specific promoters, and their editing stability can be effectively ensured by controlling the number of tandem target sites and optimizing the use of endogenous RNA processing machinery.

Furthermore, the introduction of epigenetic modification enzymes provides a new approach to overcoming the bottleneck of editing efficiency. Recent studies have shown that co-expressing the human RNA demethylase hFTO (Human Fat Mass and Obesity-Associated Protein) in CRISPR vectors can specifically remove m⁶A methylation modifications from sgRNAs and target mRNAs. This modification can increase the editing efficiency of multiple low-efficiency editing sites (e.g., *OsPDS* in rice, *AtPDS3* in *Arabidopsis thaliana*) by 2–3 times, and has been successfully applied to multiplex genome editing in the soybean hairy root system (Bai et al. 2024).

## Research advances in multiplex genome editing for crop breeding

**Fig. 2:**
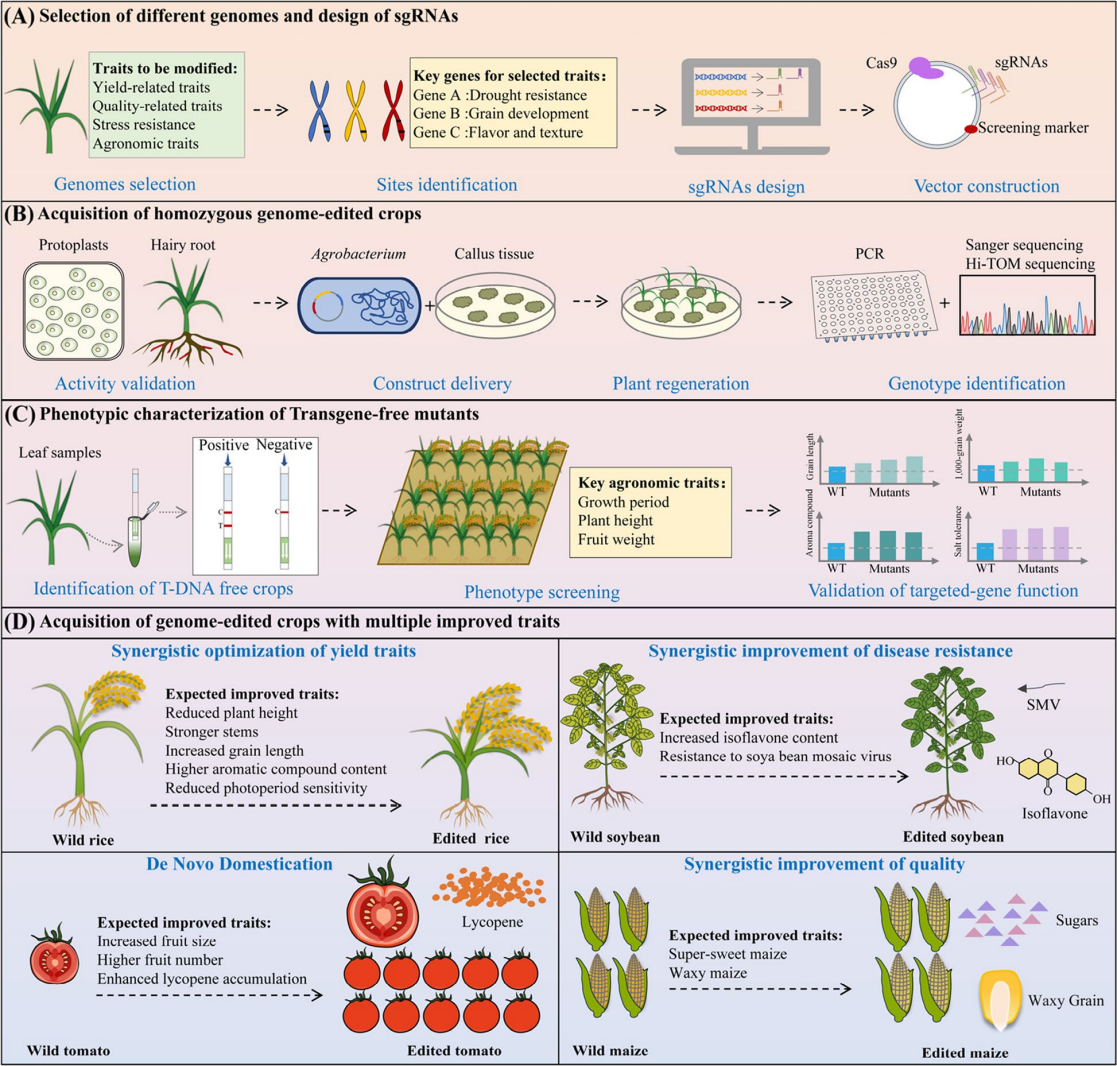
Technical workflow of multiplex genome editing for crop improvement. **A** Target screening and vector construction: Based on target agronomic traits, candidate genes are identified through genome-wide screening. Highly specific sgRNAs are designed and validated in silico, followed by cloning into a CRISPR/Cas expression vector. **B** Genetic transformation and mutant identification: The editing vector is delivered into plant cells via *Agrobacterium*-mediated transformation or other methods, followed by plant regeneration through tissue culture. Homozygous edited mutants are identified using PCR and sequencing. **C** Transgene-free screening and trait evaluation: Plants free of T-DNA integration are selected. Key agronomic traits are evaluated under field or controlled conditions, and editing effects are validated through statistical comparison with wild‑type controls. **D** Trait pyramiding and germplasm development: Multiple genes are simultaneously edited in a single generation to achieve trait stacking, enabling rapid development of new germplasm with superior comprehensive traits

In the process of modern agricultural development, the synergistic optimization of crop traits is crucial for variety improvement and meeting diverse consumer demands. By simultaneously targeting multiple functional genes, multiplex gene editing provides a revolutionary solution for the pyramiding of multiple crop traits. The steps of multiplex genome editing in crops are illustrated in Fig. 2. As shown in Table 2, examples of CRISPR/Cas9-based synergistic improvement of multiple crop traits in recent years are listed. Single vectors now carry 2–16 sgRNAs, a breakthrough that supports the development and application of crop gene editing.

**Table 2:** Examples of multiplex genome editing for crops

Crop	Target gene(s)	Plant phenotype	Reference
Rice	*PIN5b, GS3, MYB30*	High yield, cold tolerance	(Zeng et al. 2020)
Rice	*GS3, TGW3, GW8*	High yield, quality	(Huang et al. 2024)
Rice	*P450, BADH2*	Increased yield, fragrance	(Usman et al. 2020)
Rice	*Bsr-d1, Pi21, ERF922*	Resist blast, bacterial blight	(Zhou et al. 2022)
Rice	*Pi21, Xa5, BADH2*	Fragrance, disease resistance	(Zhang et al. 2024)
Rice	*Ghd7, DTH8, DTH7, *etc	Early maturity, better architecture	(Wei et al. 2024)
Rice	*Hd2, Ghd7, DTH8, *etc	Early flowering, disease resistance	(He et al. 2024)
Rice	*SD1, GS3, Hd1*, etc	Improved plant type, higher yield	(Hao et al. 2025)
Wheat	*Gli-γ, Gli-ω*	Low gliadin, allergens	(Sánchez-León et al. 2024)
Soybean	*NIC, RIC1, RIC2*	High yield, protein	(Zhong et al. 2024)
Soybean	*F3H1, F3H2, FNSII-1*	More isoflavone, disease resistance	(Zhang et al. 2020)
Soybean	*EIL3, EIL4, EIN2L*	Early flowering, high yield	(Cheng et al. 2023)
Soybean	*RS2, RS3*	Low RFO, high sucrose	(Cao et al. 2022)
Tomato	*PSY1, MYB12, SGR1*	Customized carotenoid, flavonoid, chlorophyll	(Yang et al. 2023)
Tomato	*SP, SICLV3, *etc	Larger fruits, more vitamin C/lycopene	(T. Li et al. 2018a)
Maize	*SH2, WX*	Sweet and waxy corns	(Dong et al. 2019)

### Synergistic improvement of disease/insect resistance and abiotic stress tolerance traits

In modern agriculture, crops are frequently exposed to both biotic and abiotic stresses, and traditional single-gene improvement approaches struggle to address these complex environmental challenges (Pandey et al. 2017).

In the field of biotic stress management, the evolution of pest and disease resistance and the homogeneity of resistance genes have long posed significant challenges in breeding. Traditional disease resistant breeding relies heavily on single major effect genes, which can temporarily protect against specific pathogens but are prone to resistance breakdown due to pathogen mutations and often fail to confer broad-spectrum resistance. Emerging studies have demonstrated that multiplex genome editing can construct multi-layered immune defense systems in crops by simultaneously targeting multiple disease-related genes (Ishikawa et al. 2022; S. Li et al., 2022; Yu Lu et al., 2021; Lyu et al. 2022; Mubarik et al. 2021). In rice breeding, simultaneous targeting of three key disease resistance genes (*Bsr-d1*, *Pi21*, *ERF922*) using three sgRNAs successfully developed new varieties with significant resistance to both rice blast and bacterial blight. Field trials showed that their resistance durability was 2–3 years longer than that of single-gene improved varieties (Zhou et al. 2022). The multi-gene synergistic strategy not only broadens the resistance spectrum but also reduces the risk of pathogen resistance evolution by disrupting multiple infection pathways.

The complexity of abiotic stress lies in the diversity of stress factors (drought, salinity, extreme temperatures, etc.) and the intersectionality in gene regulatory networks. Traditional stress resistant breeding often encounters the bottleneck that stress resistance is negatively correlated with yield. Overexpression of a single stress-related gene may enhance stress tolerance but at the expense of growth and development efficiency. For example, rice overexpressing the drought resistant gene *DREB1A* exhibits improved drought tolerance but reduced plant height and seed-setting rate (Kasuga et al. 1999). Multiplex genome editing can balance stress tolerance and agronomic traits by synergistically optimizing the functions of multiple genes (M. Chen et al., 2021; J. Li et al., 2021). In tomato salt-tolerance breeding, different functional domains of the *HyPRP1* protein were targeted using four sgRNAs. Edited plants showed over 40% improvement in salt tolerance and simultaneously enhanced cross-resistance to drought and cold through activation of downstream osmotic adjustment and antioxidant genes (Tran et al. 2021). Notably, synergistic improvement in rice cold tolerance and yield was achieved by precisely editing three genes (*PIN5b*, root development; *GS3*, grain shape regulation; *MYB30*, cold response) using six carefully designed sgRNAs. Edited plants showed only a 3.2% yield loss under cold stress, significantly lower than the 15%–20% loss in traditional cold-tolerant varieties (Zeng et al. 2020). Additionally, crops are highly sensitive to temperature fluctuations. Regulating genes involved in cold and heat responses, such as *HSPs* (heat shock proteins), *CBFs* (C-repeat binding factors) and so on, can effectively enhance plant stress tolerance (Chinnusamy et al. 2007; Ren et al. 2021; B. Wang et al., 2020; Zhou et al. 2019). These examples highlight the unique advantages of multiplex genome editing in addressing complex environmental challenges.

### Synergistic optimization of multiple genes for yield traits

Crop yield is regulated by polygenic networks, encompassing processes such as biomass allocation, photosynthetic efficiency, and grain development. Due to the difficulty in breaking genetic linkages between traits, traditional breeding methods often fail to achieve the optimal combination of these key factors. Multiplex genome editing provides a precise tool for the synergistic optimization of yield-related genes (Xu et al. 2016; Zhou et al. 2019), with its unique advantage being the ability to create favorable trait combinations that are rare or even non-existent in nature. For example, the *PIN5b* (regulates root development), *GS3* (controls grain length), and *MYB30* (regulates cold tolerance) genes in rice were simultaneously edited, achieving simultaneous improvement in yield and stress tolerance (Zeng et al. 2020). High-yielding hybrid rice with slender grain shape was developed by targeting the *GS3*, *TGW3*, and *GW8* genes (Huang et al. 2024). Additionally, 8 sgRNAs were designed to target 3 cytochrome *P450* homologs and the yield-related gene *BADH2*, which not only significantly increased rice yield but also endowed rice grains with a fragrant aroma (Usman et al. 2020). Flowering time is a key agronomic trait for soybean yield and adaptability. Rice with multiple favorable traits (early flowering, disease resistance, and fragrance) was successfully developed by targeting three flowering-time genes (*Hd2*, *Ghd7*, and *DTH8*), the rice blast resistance gene (*Pi21*), and the fragrance gene (*BADH2*) using 5 sgRNAs in tandem driven by a single promoter (He et al. 2024). This indicates that multiplex genome editing has been widely applied in rice breeding.

In other major crops, multiplex genome editing technology also demonstrates strong application potential. In soybean breeding, the ideal combination of early flowering and high yield has been achieved by knocking out *EIL3*, *EIL4*, and *EIL2L* in the ethylene signal transduction pathway (Cheng et al. 2023). A particularly groundbreaking study was conducted, in which the *RIC1a* and *RIC2a* genes were edited (Zhong et al. 2024). This successfully broke the negative correlation between yield and protein content, achieved synergistic optimization of carbon–nitrogen metabolism, and developed new high-yield, high-protein soybean varieties. Additionally, in tomato breeding, 8 sgRNAs encoded by a single transcript were used to target 6 key agronomic trait genes, enabling rapid domestication of wild tomatoes and obtaining new germplasm with increased yield, larger fruits, and higher lycopene content (T. Li et al. 2018a).

### Synergistic improvement of crop quality and other traits

Multiplex genome editing provides a precise tool for improving crop nutritional quality. Quality improvement mediated by multiplex genome editing is generally observed in nutritional components, taste and flavor, appearance characteristics, and processing properties (Liu et al. 2021).

#### Multi-dimensional improvement of nutritional components

Crops are rich in various nutrients, and increasing the content ratio of their nutritional elements aligns with modern consumers' pursuit of healthy food. Currently, the optimization of the content levels of multiple nutritional elements in crops (e.g., isoflavones, carotenoids) has been successfully achieved through gene editing technology (X. Li et al., 2018; Zhang et al. 2020). In wheat quality improvement, *Pinb, Waxy, Ppo, and Psy* related to grain quality were targeted, and wheat grain hardness, starch quality, and carotenoid content were simultaneously optimized (Zhang et al. 2021). Meanwhile, reducing the content of anti-nutritional factors using multiplex genome editing technology is also a direction in crop improvement (Jouanin et al. 2019; Kim et al. 2024; Lin et al. 2024, 2023; Wang et al. 2023).

#### Synergistic improvement of flavor and appearance traits

The taste and flavor of crops are also important factors affecting their market acceptance. Multiplex genome editing can enhance crop flavor characteristics by removing unpleasant volatile substances and promoting the synthesis of aroma compounds. For example, editing the *BADH2* gene in rice endowed rice with a fragrant, popcorn-like aroma (Usman et al. 2020; Zhang et al. 2024). In soybeans, three lipoxygenase genes (*LOX1*, *LOX2*, *LOX3*) were knocked out, and new germplasm without beany flavor was successfully developed (J. Wang et al., 2020). Additionally, improving the shape and size of crops is of great significance. In existing studies, breeding for rice appearance characteristics is relatively common; for instance, optimizing rice appearance quality by regulating grain length and weight (Huang et al. 2024; Xu et al. 2016). A multiplex genome editing-based rapid directional improvement (MRDI) strategy was established, which generated complete diversity of 12 key agronomic genes in rice to directionally improve its growth duration and plant architecture. This resulted in ideal plants with early heading date, reduced plant height, and more efficient panicles (Wei et al. 2024). Furthermore, researchers also improve crop color by regulating pigment synthesis genes to make crops more attractive in the market. Taking tomatoes as an example, their fruit color is determined by pigments such as carotenoids, flavonoids, and chlorophyll. Multiplex genome editing was used to target and knock out key genes (*PSY1*, *MYB12*, *SGR1*) to achieve rapid customization of tomato fruit color (Yang et al. 2023).

#### Improvement of processing properties

Soybean is one of the sources of vegetable oil, and its unsaturated fatty acids exhibit strong oxidative stability and thermal stability. Increasing the content of unsaturated fatty acids can improve the utilization of soybean oil and is beneficial to human health (Lakhssassi et al. 2017). The fatty acid desaturase 2 (*GmFAD2*) gene can convert monounsaturated oleic acid (C18:1) into polyunsaturated linoleic acid (C18:2), thereby regulating the content of monounsaturated fats in soybean seeds. High-oleic acid soybeans were successfully created by knocking out two key fatty acid desaturase genes, *GmFAD2–1A* and *GmFAD2–1B* (Do et al. 2019). Building on this, researchers further achieved the effect of stacked knockout of lipoxygenase (*LOX*) genes, successfully developing new high-oleic acid soybean germplasm without beany flavor, which further improved the processing quality and market value of soybeans (Zi-ying et al. 2023). Multiplex genome editing goes beyond optimizing fatty acid profiles and flavor—it also enables tailored enhancement of functional properties in soybean proteins. The approach involves combining two multiplex-edited soybean varieties, each targeting unique gene clusters associated with protein structure (e.g., storage protein genes including *Glycinin* and *β-Conglycinin*, as well as genes related to processing traits). Through this strategy, researchers can precisely modulate key protein functionalities such as emulsifying activity, gelation behavior, and solubility (Bai et al. 2022).

### Multiplex genome editing technology enables de novo domestication in crops

Traditional domestication has directionally optimized core agronomic traits such as crop yield and plant architecture through long-term artificial selection, but it has also inevitably led to a narrow genetic base and the loss of naturally superior traits including stress resistance and disease resistance, thus forming a domestication bottleneck (Van De Wouw et al. 2010). Multiplex genome editing technology has effectively enabled the de novo domestication of wild crop relatives by directly targeting domestication genes, preserving beneficial crop traits and expanding the gene pool. At present, this technology has achieved substantial breakthroughs in de novo domestication research of various crops, and has demonstrated remarkable advantages especially in the improvement and domestication of characteristic germplasm (T. Li et al. 2018b; Zsögön et al. 2018).

In the research on rice de novo domestication, researchers established the first technical system for the de novo domestication of wild allopolyploid rice (*Oryza alta*). By editing the homologous genes in the *PPR1* gene that control seed shattering, awn length, plant height, grain length, stem thickness and growth period, various gene-edited materials were successfully created with reduced seed shattering, shortened awn length, dwarfed plant height, increased grain length, thickened stems and shortened heading date to varying degrees (Yu et al. 2021). Sea Rice 86 (SR86), a high-quality salt-tolerant rice derived from ancient indica rice, possesses excellent salt tolerance, yet its production and application are limited by wild traits such as excessive plant height, easy seed shattering and low yield. Researchers utilized this technology to synchronously edit 13 key agronomic trait genes of Sea Rice 86 (Hao et al. 2025). Through the construction of a multi-gene editing vector and a single genetic transformation, the homozygous line SR86M with the optimal editing effect was screened out from the T_0_ generation plants. While maintaining salt tolerance comparable to that of Sea Rice 86, this line successfully improved 7 important agronomic traits including plant height, plant architecture, grain shape, yield components and photoperiod sensitivity, providing important practical insights for the rapid domestication of characteristic germplasm and the cultivation of varieties with excellent comprehensive traits.

## Challenges and opportunities of multiplex gene editing in crop traits pyramiding

### Synergistic risks and improvement potential of multiplex gene editing

The core challenge of multiplex genome editing technology in improving complex crop traits lies in the complex interaction relationships between genes. Different gene combinations may produce various interaction patterns such as synergistic, antagonistic, or independent effects, which profoundly affect the outcome of trait improvement.

In terms of synergistic improvement, multiple genes editing has demonstrated significant potential. For instance, in tomato quality improvement, simultaneous knockout of *LCY*, *SGR1*, and *BLC1* can block carotenoid branch metabolism, leading to a 5 times increase in lycopene accumulation (X. Li et al., 2018), which demonstrates the effective regulation of metabolic pathways by multiple genes synergistic editing. Additionally, knocking out the *RIC* gene in soybeans can increase nodule number to enhance soybean yield (Bai et al. 2020). Meanwhile, synergistic editing of *NIC* (nitrogen absorption) and *RIC* (protein synthesis) genes has broken the negative correlation between protein content and yield (Zhong et al. 2024). When genes belong to different pathways, such as the co-editing of rice fragrance gene *BADH2* and rice blast resistance gene *Pi21*, improvement of independent trait can be achieved without significant interaction effects between genes (Zhang et al. 2024).

However, the complexity of gene interactions may also trigger antagonistic effects. In rice high-yield breeding research, simultaneous editing of *GS3* (grain length), *GW2* (grain width), and *Gn1a* (grain number) can significantly improve crop yield. However, the loss of *Gn1a* function leads to overactivation of the cytokinin signaling pathway, causing an abnormal increase in tiller number and thereby affecting plant lodging resistance (Zhou et al. 2019). Nevertheless, such issues can be alleviated by optimizing gene combinations. Simultaneous editing of rice high-yield gene *Ghd7*, early-flowering gene *DTH8*, and drought-tolerant gene *OsNAC006* can effectively balance yield and stress-resistant traits (He et al. 2024). It is evident that multiple traits pyramiding breeding requires full dissection of multiple genes interaction networks and dynamic balance of the correlation effects between traits (Hao et al. 2025).

### Cas12a: an advanced nuclease tool for multiplex editing

In the field of multiple gene pyramiding breeding, traditional CRISPR/Cas9 systems have significant limitations, which restrict their application and development. For example, tandem expression of multiple sgRNAs requires the introduction of a large number of repeated promoters, which easily triggers vector homologous recombination or RNA interference, leading to a sharp decline in the simultaneous assembly efficiency of more than 4 sgRNAs (Ding et al. 2020). The iterative upgrading of gene editing tools provides key technical support for improving complex crop traits, and novel nucleases represented by Cas12a (Cpf1) possess distinct advantages for multiplex editing applications.

Unlike Cas9, Cas12a utilizes shorter CRISPR RNA (crRNA) with minimal repeat sequences (Zetsche et al. 2015). The structural difference reduces sequence redundancy in tandem arrays by over 50%, significantly lowering vector recombination rates (Wu et al. 2018). For instance, the recombination rate for the 6-sgRNA array of Cas9 is approximately 35%, while that for the 8-crRNA array of Cas12a is below 10% in rice (Hu et al. 2020). Furthermore, Cas12a possesses intrinsic RNase activity, enabling autonomous processing of multi-transcript crRNA arrays into individual functional crRNAs without requiring exogenous cleavage elements needed by the Cas9 system (J. Li et al., 2022). This feature enables the stable serial assembly of 6–8 crRNAs within a single vector, achieving effective protein-coding gene knockout editing across multiple crops. In recent years, engineered Cas12a ortholog have achieved efficient multi-gene modification in both monocot and dicot crops. By optimizing the crRNA scaffold and adding a nuclear localization signal (NLS), efficient editing of 8 loci (*GhPGF*, *GhCLA1*, *GhFAD*, *GhTAC1*, *GhMYB25*-like*)* in cotton has been achieved, demonstrating the broad applicability of engineered Cas12a variants in multiplex gene editing (Hui et al. 2024). In addition, the Mb3Cas12a (Cas12a ortholog) STU system has been successfully developed to optimize PAM compatibility and temperature adaptability (Liu et al. 2025). It has been applied to single-gene and multiplex genome editing in rice, maize, and tomato. This system not only recognizes the canonical TTTV PAM sites but also achieves an editing efficiency of up to 100% at VTTV PAM sites. Moreover, it maintains high editing activity even at low temperatures, overcoming the limitations of traditional LbCas12a, such as a narrow PAM recognition range and strong temperature dependence. For multiplex editing, the Mb3Cas12a STU system enables multi-gene editing of three quality-related genes in tomato, generating mutants with increased lycopene content and a co-editing efficiency of 4.0% for the three target genes.

Despite the continuous optimization of novel nuclease tool performance, editing efficiency remains a core challenge restricting large-scale multi-gene pyramiding. Currently, the simultaneous editing efficiency of 3–5 genes can be maintained at 50%–70%, but when the number of targets exceeds 8, the efficiency often drops to below 30% (Cheng et al. 2023; Usman et al. 2020; Wei et al. 2024; Zhang et al. 2021), highlighting the need for both tool upgrading and auxiliary design strategies.

### Intelligent design strategies for precision multiplex editing

To address the efficiency attenuation of large-scale multiplex editing and improve the precision of trait pyramiding, multi-omics-assisted design and artificial intelligence-driven intelligent strategies have promoted the transformation from empirical target selection to rational design.

Multi-omics data integration provides a systematic molecular basis for multiplex editing target screening. By integrating transcriptomic, metabolomic, and proteomic data, the impact of gene editing on metabolic networks can be systematically dissected (Bryant et al. 2022). Meanwhile, machine learning models can perform deep learning on large-scale editing data sets to predict the optimal sgRNA combination, target site accessibility and editing efficiency, further improving the success rate of multiplex editing (Zhang et al. 2023). Notably, the potential of Large Language Models (LLMs) in optimizing sgRNA sequence design for multiplex editing has emerged as a cutting-edge research direction in this field. CRISPR-GPT, an LLM agent specially developed for the automated design of gene-editing experiments, can integrate multi-dimensional biological information including genomic sequence characteristics, epigenetic modification patterns and Cas protein binding preferences. It breaks through the limitations of traditional empirical sgRNA design and realizes the intelligent optimization of multi-target sgRNA sequences with both high targeting efficiency and low off-target risk through automated sequence analysis and rule adaptation, thus effectively improving the design efficiency and precision of multiplex editing systems (Qu et al. 2024). AlphaFold, as an intelligent protein structure prediction tool, plays a pivotal guiding role in multiplex gene editing (Bryant et al. 2022). First, it can simulate the three-dimensional structures and conformational changes of protein complexes following multiplex gene editing, and predict whether the edited target proteins maintain normal protein–protein interactions and functional stability, avoiding invalid editing caused by structural damage of key functional proteins. For example, through AlphaFold 2-guided bespoke gene editing, a series of mutations are generated for target genes and their structures are predicted; mutations similar to known favorable alleles (high-oil type) are then screened out via structural clustering, and the required mutation types are created using gene editing techniques (Wang et al. 2025). These results demonstrate that AlphaFold 2-guided editing enables the precise design of function-optimized alleles, significantly improving the directional efficiency of multiplex editing. With the advancement of AlphaFold to its third generation, prediction accuracy has been improved and the tool is now capable of integrated protein complex prediction, yet relevant research in the plant field remains scarce. In rice drought-tolerance breeding, AlphaFold 3 can predict significant epistatic interactions among different allelic combinations of the *OsMYB2*, *OsGH18* and *OsCAD3* genes; the functional module formed by these three genes coordinately regulates lignin accumulation and drought tolerance in rice (Jeppesen and André, 2023). For multi-trait pyramiding, this finding indicates the predictability of combinations of different alleles of various genes across different traits. Under the guidance of AlphaFold, the selection of optimal target combinations is of great importance for precision breeding.

In addition to protein-coding gene editing, the functional dissection and precise editing of non-coding regions (promoters, untranslated regions, etc.) have become a key area for future breakthroughs in multiplex editing. Although existing studies have focused on non-coding region editing for crop trait improvement (Tan et al. 2023; Wang et al. 2024; H. Zhang et al., 2018; Zhou et al. 2024), there is still a lack of systematic understanding of the complex relationship between sequence higher-order structures and regulatory elements, making it difficult to accurately predict their impact on trait formation. The development of high-resolution functional annotation technologies for non-coding regions and customized editing strategies will further expand the application scope of multiplex gene editing in crop breeding and provide new targets for complex trait improvement. Thus, developing tools to predict protein function is crucial for enabling editing across multiple non-coding regions.

### Next-generation precision editing technology: prime editing

Notably, multi-site precision editing based on Prime Editing(PE) is emerging as a key future trend in polygenic trait aggregation for crop breeding (Anzalone et al. 2019; Vats et al. 2024). Most beneficial agronomic alleles do not originate from complete gene knockout, but are driven by synergistic variations of single nucleotide polymorphisms (SNPs), small indels, and associated genetic elements (Bradbury et al. 2005; Y. Z. Chen et al., 2021; Lakhssassi et al. 2021; Wang et al. 2021).

The unique advantage of PE technology lies in its ability to precisely introduce such complex genetic variations without generating DSBs. This avoids the random indels and genetic instability associated with traditional CRISPR-Cas9, making PE an indispensable tool for improving complex traits in crops (Anzalone et al. 2019; Lin et al. 2020). Breakthroughs include the MAMPE system in rice, which achieved simultaneous editing of multiple key endogenous genes including the grain length gene *GS3*, the grain weight gene *GW2*, and the aroma gene *BADH2*. T_0_ plants demonstrated 28.6% efficiency for dual-site editing and 7.1% for quadruple-site editing, verifying the feasibility of PE technology for multi-gene stacking in crops (Gupta et al. 2024). In dicot crops, prime editing technology still faces the challenge of low efficiency in single-gene precise editing. An ultra-efficient prime editing system (UtPE) developed in tomato has broken the bottleneck of low PE efficiency in dicot crops (Van Vu et al. 2026). It adopts evolved PE6 variants to enhance editing enzyme activity, matches optimized aepegRNAs to improve targeting accuracy, introduces RNA chaperone proteins to stabilize pegRNAs, and uses geminivirus replicon vectors to amplify editing signals. This integrated strategy increases the single-target editing efficiency of T_0_ generation tomato plants to 87.5%. More importantly, the efficiency of simultaneous multi-gene editing is not significantly different from that of single-target editing. The system has successfully achieved concurrent precise editing of the tomato jointless trait gene (*SlMBP21*) and herbicide resistance gene (*SlEPSPS1*), obtaining elite lines with stably heritable agronomic traits. In addition, the developed flanking-nicks prime editor (FLICK-PE) system significantly improves the editing efficiency of dicot crops such as soybean and tobacco by introducing double nicks on both sides of the non-editing strand to regulate the DNA mismatch repair pathway, providing new technical support for multiplex gene editing in dicotyledonous plants (Bai et al. 2025).

Nevertheless, PE-based multi-site editing technology remains in its infancy in crops, facing multiple challenges including efficiency, delivery and scalability (Jin et al. 2023; Yuming Lu et al., 2021a; Perroud et al. 2022). Future optimization should focus on enhancing stability by modifying pegRNA scaffolds and driving reverse transcriptase expression using tissue-specific RNA polymerase II promoters (Lin et al. 2020; Nelson et al. 2022). Integrating these strategies will significantly boost multi-site editing efficiency, ultimately enabling precise customization of complex beneficial alleles and propelling crop breeding from single-gene improvement toward synergistic multi-trait optimization.

### Exploration of delivery systems for multiplex genome editing

Large-scale application of multiplex genome editing in crops is highly dependent on efficient delivery systems. Agrobacterium-mediated transformation and particle bombardment remain the mainstream technical approaches to date. However, in the face of the demands for delivering multiple targets and multiple components, their inherent limitations and the bottlenecks in in vitro plant transformation have become increasingly prominent. Although Agrobacterium-mediated transformation offers the advantages of high efficiency, high throughput and facile low-copy integration, it suffers from a narrow host range and limited T-DNA carrying capacity, which precludes the efficient delivery of large vectors containing multiple editing cassettes. In addition, the subsequent T-DNA segregation is cumbersome, and the challenge of transforming recalcitrant crops has not yet been overcome (Luo et al. 2025).Particle bombardment can deliver larger DNA fragment vectors and has a broader host range, making it suitable for crop species that are difficult to infect with Agrobacterium. Nevertheless, it is plagued by low transformation efficiency, a high rate of random integration of exogenous DNA, and a tendency to induce aberrant gene expression. Despite the improvement of gene editing efficiency from 4.0% with traditional methods to 8.7%–9.3% via tool optimization (e.g., flow-guiding barrel modification), this approach still cannot meet the requirements of large-scale application for multiplex genome editing (Thorpe et al. 2025). Furthermore, both methods rely on in vitro tissue culture with cumbersome operational procedures, which further restricts their large-scale popularization.

To break through the aforementioned bottlenecks, novel delivery systems based on viral vectors and nanomaterials have become a research hotspot, providing a new avenue for tissue culture-independent genome editing (Steinberger and Voytas 2025; Das et al. 2026). Among these, viral delivery systems exhibit enormous application potential by virtue of their unique advantages. Notably, the virus-induced genome editing (VIGE) technology mediated by RNA viral vectors involves inserting CRISPR gene-editing components into viral genomes and efficiently delivering editing tools into plant cells via viral infection to achieve targeted genome editing (Ma et al. 2020). Compared with Agrobacterium-mediated transformation and particle bombardment, viral vector delivery systems first mitigate the risk of random mutations caused by exogenous DNA integration. Secondly, engineered viral vectors enable the delivery of editing components through both tissue culture-independent and tissue culture-dependent approaches, greatly simplifying the operational procedures.

More crucially, viral vectors exhibit remarkable superiority in the targeted delivery of multiple editing targets. For tissue culture-independent plants, positive-sense RNA viruses such as Barley stripe mosaic virus (BSMV) are the primary tools employed. Using the BSMV-sgRNA system to deliver sgRNAs targeting the *PDS*, *GW2* and *GASR7* genes into wheat varieties stably expressing Cas9, the maximum editing efficiency of dual genes in the M_0_ generation reached 47.3%; for triple-gene editing, simultaneous mutation of two genes was achieved with an efficiency ranging from 11.1% to 50.0%. Impressively, 83.3% to 91.7% of the multiplex gene-edited mutants in the M_1_ generation were virus-free (T. Li et al., 2021). In addition, tissue culture-dependent VIGE technology mainly relies on negative-sense RNA viruses (e.g., SYNV). This type of system can deliver the complete CRISPR/Cas system (including Cas9 nucleases and sgRNAs) in a single round of delivery, eliminating the need for pre-creation of Cas-transgenic plants. The CRISPR-Cas9 editing component delivery system constructed on the basis of the SYNV vector can generate homozygous or tetra-allelic mutants after infecting tobacco tissues and subsequent regeneration, and the mutation efficiency of two gene targets achieved by the multiplex vector is comparable to that achieved by vectors expressing a single sgRNA (Ma et al. 2020).

However, viral vector delivery systems still have obvious application limitations: most vectors for tissue culture-independent VIGE technology have a limited loading capacity and are currently restricted to the single delivery of sgRNAs. Efficient genome editing thus still relies on the construction of transgenic plants stably expressing Cas9, which fails to completely eliminate tissue culture operations and results in poor applicability to transformation-recalcitrant crops. Only a few viral vectors can simultaneously deliver Cas proteins and sgRNA editing components, yet the edited somatic cells require induction of regeneration through tissue culture. Therefore, future research needs to focus on the exploration of efficient and compact Cas systems, as well as the modification of high-capacity viruses to deliver multi-sgRNA systems simultaneously, thereby achieving DNA-free genome editing in crops.

## Data Availability

No datasets were generated or analyzed during the current study.
